# Long covid and mental and physical health: A cross‐sectional study of adults in California

**DOI:** 10.1002/puh2.152

**Published:** 2024-01-15

**Authors:** Tyler B. Mason, Tara K. Knight, Ryan Lee, Shirin E. Herzig, Daniella Meeker, Jason N. Doctor

**Affiliations:** ^1^ Department of Population and Public Health Sciences University of Southern California Keck School of Medicine Los Angeles California USA; ^2^ Sol Price School of Public Policy University of Southern California Los Angeles California USA; ^3^ Yale School of Medicine Yale University New Haven Connecticut USA

**Keywords:** COVID‐19, fatigue, long covid, mental health, physical health

## Abstract

**Introduction:**

Most people recover from COVID‐19 infection over a short period of time, but a minority of individuals experience symptoms over a longer duration (≥28 days), termed “long covid.” The purpose of the current study was to examine differences between individuals with a long covid diagnosis (i.e., diagnosed long covid), who believe they do or might have long covid (i.e., self‐reported long covid), and people without long covid.

**Methods:**

Adults who had been diagnosed with COVID‐19 completed survey questions about COVID‐19 history, long covid, and mental and physical health. Analysis of covariance models showed an effect of long covid group (i.e., diagnosed, self‐reported, and no long covid) with anxiety, depression, physical function, fatigue, social roles/activity limitations, and pain interference.

**Results:**

Analyses demonstrated that the self‐reported long covid group had significantly greater anxiety and depression than the no long covid group. The diagnosed long covid group had significantly greater physical function problems than the no long covid group. Both diagnosed and self‐reported long covid groups had significantly greater fatigue, social roles/activity limitations, and pain interference as compared to the no long covid group.

**Conclusion:**

Overall, physical health challenges were reported by individuals with long covid, with fatigue being the most significant symptom. In addition, negative mental health was only experienced by individuals with self‐reported long covid, suggesting the importance of long covid diagnosis and treatment.

## Graphical abstract

Physical health challenges were reported by individuals with long covid, yet negative mental health was only experienced by individuals with self‐reported long covid. Findings suggest the need for more research on long covid course and interventions.

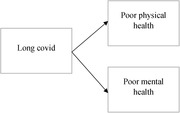

## BACKGROUND

The 2019 coronavirus (COVID‐19) pandemic resulted from the spread of severe acute respiratory syndrome coronavirus 2 (SARS‐CoV‐2) infection, which is an intense, acute illness that primarily affects the immune and respiratory systems of the body [1]. Although many individuals who contract COVID‐19 recover over a short period of time, a minority of individuals experience persistence of symptoms over a longer duration (≥28 days), termed “long covid” [2]. Evidence suggests that long covid is experienced by 5%–85% of individuals diagnosed with COVID‐19 and has a negative impact on quality of life and recovery [3, 4]. Long covid can include a range of symptoms, such as fatigue, muscle pain, weakness, mild fever, cough, breathlessness, chest pain, headaches, cognitive difficulties, tingling sensations, and skin rashes [5]. Individuals infected with SARS‐CoV‐2 who report five or more of these symptoms are more likely to develop long covid than those with fewer symptoms [5].

Women are more likely to develop long covid than men [6]. Moreover, those with long covid have been recorded to have a slower recovery from certain symptoms, including breathlessness and fatigue [7]. The most common symptoms that are early signs of long covid include fatigue, shortness of breath, hoarse voice, myalgia, and migraines [5]. Moreover, fatigue is the most common long covid symptom and typically lasts beyond the initial infection, especially for those hospitalized [7]. Long covid has been shown to also have significant impacts on mental health, with some positing that poor mental health is a symptom of long covid [8]. Some of the common mental health issues that COVID‐19 survivors have reported include post‐traumatic stress disorder, depression, and anxiety disorders [1, 9]. Overall, reviews have shown that long covid affects a variety of symptoms, including mental health, gastrointestinal, cardiopulmonary, and neurological, without a clear etiological explanation [10, 11]. However, with SARS‐CoV‐2 being a novel virus, the long‐term effects of COVID‐19 on survivors are still being uncovered [1].

Although there has been a great deal of research on the COVID‐19 pandemic and mental and physical health, less is known about the mental and physical health correlates associated with long covid, and it has been particularly difficult to characterize long covid due to the subjective nature of symptoms. The purpose of the current study was to examine differences between individuals with a long covid diagnosis (i.e., diagnosed long covid), who believe they do or might have long covid (i.e., self‐reported long covid), and people without long covid. Some research has proposed that long covid may be due to subjective, biopsychosocial factors related to the COVID‐19 pandemic and infection rather than the virus itself, such as distress about the COVID‐19 pandemic [12]. As such, COVID‐19 distress was included as a covariate in analyses. In addition, the COVID‐19 pandemic and long covid affected many demographic groups differently [6, 13, 14]; thus, sex, age, race/ethnicity, and body mass index (BMI) were controlled for in analyses. Disordered eating was included as a mental health outcome. To our knowledge, the association between long covid and disordered eating has not been tested. Yet, it was important to include disordered eating given that the COVID‐19 pandemic had a negative effect on eating disorders [15, 16]; previous studies have shown associations between disordered eating and physical symptoms and chronic illnesses [17, 18, 19]; and disordered eating is often omitted from studies on mental health [20]. It was hypothesized that long covid diagnosis and self‐reported long covid would be associated with poorer mental and physical health compared to no long covid.

## METHODS

### Participants and procedures

Adults who had been previously infected with SARS‐CoV‐2 were invited to participate in an online survey‐based study. Participants were recruited online through the University of Southern California (USC) COVID‐19 Biospecimen Repository (https://sc‐ctsi.org/about/covid‐19‐biorepository) and the USC Clinical Data Warehouse. All participants visited a USC healthcare center in Los Angeles County in the United States within the past 3 years. Emails were obtained from medical records, and invitations were sent to patients who met study eligibility criteria. Data collection took place from March 2023 to June 2023. Individuals with records of a COVID‐19 infection were contacted, and past COVID‐19 infection was confirmed with a COVID‐19 history questionnaire. Potential participants received an invitation and custom REDCap link to enroll in the study. Informed consent was obtained through e‐consent in REDCap. Participants then completed an online survey, including questionnaires on demographics, COVID‐19 symptoms and psychosocial impact, mental health, and quality of life. All study procedures were reviewed and approved by the USC Institutional Review Board (HS‐20‐00793).

### Measures

#### Long covid

Participants were asked if they have been diagnosed with “long covid.” They were provided a definition of long covid as “continuing to have signs and symptoms four weeks after the diagnosis of SARS‐Cov‐2 infection that are not explainable by other causes.” If they answered “no,” participants were asked if they believe they have long covid with options “yes,” “no,” and “maybe.” Long covid was trichotomized as “yes, diagnosed,” “yes/maybe self‐reported,” and “no.”

#### Physical and mental health

The Patient‐Reported Outcomes Measurement Information System (PROMIS) 29 Profile v2.0 [21] includes 29 items assessing various aspects of physical and mental health. The subscales include physical function (e.g., “Are you able to do chores such as vacuuming or yard work?”), anxiety (e.g., “My worries overwhelmed me”), depression (e.g., “I felt worthless”), fatigue (e.g., “I feel fatigued”), sleep disturbance (e.g., “I had a problem with my sleep”), ability to participate in social roles and activities (e.g., “I have trouble doing all of my regular leisure activities with others”), and pain interference (e.g., “How much did pain interfere with your day‐to‐day activities?”) in the past 7 days. Each subscale includes four items; one item measuring pain intensity was not used. Items are rated on a 5‐point scale. Cronbach's alpha for the seven PROMIS subscales ranged from 0.84 to 0.97.

#### Eating disorder symptoms

The Eating Disorder Examination Questionnare‐12 was used to assess eating disorder symptoms in the past week and has been validated in previous research [22]. The scale includes 12 items that ask about eating disorder behaviors (e.g., binge eating and compensatory behaviors) and cognitions (e.g., weight and shape concerns). Ten items were rated with a scale from 1 (*0 days*) to 4 (*6–7 days*), and two items were rated with a scale from 1 (*not at all*) to 4 (*markedly*). The Cronbach alpha was 0.87.

#### COVID‐19 distress

The Pandemic‐Related Traumatic Stress Scale assessed distress stemming from the COVID‐19 pandemic [23]. The scale includes nine items based on Diagnostic Statistical Manual 5 criteria for acute stress disorder (e.g., “Since becoming aware of the COVID‐19 outbreak, how often have you felt in a daze?”). Items were rated on a scale from 1 (*not at all*) to 5 (*very often*). Cronbach's alpha was 0.89.

#### Demographics

Participants self‐reported their sex, age, race, ethnicity, height, and weight. BMI was calculated with self‐reported height and weight.

### Statistical analyses

Data analyses were conducted in SPSS v 28.0 (IBM). The expectation–maximization algorithm [24] was used to impute missing data for covariates; participants missing long covid and all outcome data were removed from analyses. Analysis of variance and multinomial logistic regressions were used to examine differences between long covid group (i.e., diagnosed, self‐reported, and no) on demographic variables and COVID‐19 distress. Bivariate correlations were also run among continuous variables. Analysis‐of‐covariance (ANCOVA) models were calculated to examine the effect of long covid group in relation to mental and physical health. Covariates were sex, age, race, ethnicity, BMI, and COVID‐19 distress. Significant main effects for long covid groups were followed up with post hoc comparison tests.

## RESULTS

Assuming *α* = 0.05, *β* = 0.20, a medium effect size (*f* = 0.25), and three covariates, power analysis showed that *N* = 64 per group (individuals with vs. without long covid) would be needed to obtain sufficient power to examine group differences in physical and mental health. However, because we did not know how many people would exhibit long covid, we collected data from a much larger group of patients. A total of 298 adults began the survey. There were 30 participants who did not provide data on long covid or any dependent variables, leaving a sample size of 268 for analyses. Most participants had complete data for all covariates (73%), with 94% of covariate values complete for the dataset. Sex at birth was 71% female and 29% male. The sample was primarily heterosexual (88%) and 12% non‐heterosexual. Regarding ethnicity, the sample was 38% Hispanic and 62% non‐Hispanic. Regarding race, the sample was 77% White, 5% Native American, Hawaiian, or Pacific Islander, 8% Asian, 6% Black or African‐American, and 4% Multiracial. The mean age was 49.48 (*SD* = 12.58), and the mean BMI was 28.63 (*SD* = 6.21).

There were 146 participants with no long covid (54.5%), 58 who reported being diagnosed with long covid (21.6%), and 64 who self‐reported that they have or might have long covid (23.9%). The three groups did not differ on sex, race, BMI, or age (*p* > 0.05), but they did differ on ethnicity (*p* = 0.01) and COVID‐19 distress (*p* < 0.001). Hispanic adults had higher rates of self‐reported long covid compared to non‐Hispanic adults (*OR* = 2.46, *p* = 0.01); there were no differences in diagnosed long covid. Diagnosed long covid (*M* = 2.88, *SE* = 0.10) and self‐reported long covid (*M* = 2.79, *SE* = 0.10) had significantly greater COVID‐19 distress than the no long covid group (*M* = 2.22, *SE* = 0.07); there were no differences between diagnosed and self‐reported long covid groups. Table 1 shows descriptive statistics and bivariate correlations of study variables.

**TABLE 1:** Descriptive statistics and bivariate correlations among study variables among University of Southern California Patients in Los Angeles, CA, 2023.

	*M*	*SD*	2	3	4	5	6	7	8	9	10	11
1. Age	49.48	12.58	−0.003	−0.18	−0.14^*^	−0.09	0.23^**^	−0.11	0.06	0.16^*^	−0.10	−0.15^*^
2. Body mass index	28.63	6.21	–	0.10	0.08	0.07	0.12	0.13^*^	0.15^*^	0.18^**^	0.12	0.32^**^
3. COVID‐19 distress	2.51	0.88		–	0.55^**^	0.46^**^	0.15*	0.38^**^	0.39^**^	0.27^**^	0.37^**^	0.22^**^
4. Anxiety	2.03	0.98			–	0.79^**^	0.24^**^	0.53^**^	0.47^**^	0.36^**^	0.46^**^	0.39^**^
5. Depression	1.87	1.00				–	0.24^**^	0.48^**^	0.44^**^	0.35^**^	0.36^**^	0.39^**^
6. Physical function	1.83	1.13					–	0.51^**^	0.68^**^	0.68^**^	0.34^**^	0.03
7. Fatigue	2.84	1.25						–	0.71^**^	0.60^**^	0.60^**^	0.26^**^
8. Social roles/Activity limitations	2.39	1.19							–	0.71^**^	0.49^**^	0.14*
9. Pain interference	2.27	1.23								–	0.47^**^	0.17^**^
10. Sleep	2.92	0.95									–	0.27^**^
11. Eating disorder symptoms	1.86	0.62										–

** *p* < 0.01.

* *p* < 0.05.

Table 2 shows results of the ANCOVAs. Sex was related to fatigue and pain interference with women reporting greater levels than men. Race and ethnicity were not associated with any mental or physical health variables. BMI was positively associated with greater pain interference and eating disorder symptoms. Greater COVID‐19 distress was associated with greater anxiety, depression, fatigue, social roles/activity limitations, pain interference, poor sleep, and eating disorder symptoms. Long covid group was associated with anxiety, depression, physical function, fatigue, social roles/activity limitations, and pain interference. There were no differences between the groups in sleep or eating disorder symptoms.

**TABLE 2:** Summary of models of associations of long covid with mental and physical health among University of Southern California Patients in Los Angeles, CA, 2023.

	Anxiety			Depression			Physical function			Fatigue		
	*F*	*p*	*η* ^2^	*F*	*p*	*η* ^2^	*F*	*p*	*η* ^2^	*F*	*p*	*η* ^2^
Sex	0.17	0.69	0.001	0.92	0.34	0.004	2.59	0.11	0.01	6.23	0.01	0.02
Hispanicity	0.84	0.36	0.003	0.44	0.51	0.002	0.04	0.85	0.000	2.13	0.15	0.01
Race	0.85	0.53	0.02	0.67	0.67	0.02	0.76	0.60	0.02	0.72	0.63	0.02
Age	0.54	0.46	0.002	0.01	0.91	0.000	16.31	<0.001	0.06	1.14	0.33	0.004
Body mass index	0.004	0.95	0.000	0.02	0.88	0.000	2.50	0.12	0.01	4.21	0.06	0.01
COVID‐19 distress	69.78	<0.001	0.22	46.29	<0.001	0.16	3.21	0.07	0.03	14.24	<0.001	0.05
Long Covid	6.48	0.002	0.05	6.36	0.002	0.05	4.30	0.02	0.01	36.12	<0.001	0.11
Model *R^2^*	0.36			0.27			0.02			0.28		

	Social roles/Activity limitation			Pain interference			Poor sleep			Eating disorder symptoms		
	*F*	*p*	*η* ^2^	*F*	*p*	*η* ^2^	*F*	*p*	*η* ^2^	*F*	*p*	*η* ^2^
Sex	1.42	0.23	0.01	7.81	0.006	0.03	0.51	0.48	0.002	0.22	0.64	0.001
Hispanicity	0.97	0.33	0.004	0.06	0.81	0.000	0.71	0.40	0.003	1.38	0.24	0.01
Race	1.04	0.40	0.02	0.40	0.88	0.01	1.35	0.24	0.03	0.57	0.76	0.01
Age	3.24	0.07	0.01	12.78	<0.001	0.05	0.00	0.99	0.06	2.21	0.14	0.01
Body mass index	3.75	0.05	0.02	7.14	0.008	0.03	1.75	0.19	0.007	24.14	<0.001	0.09
COVID‐19 distress	24.48	<0.001	0.09	6.19	0.01	0.02	17.62	<0.001	0.07	6.22	0.01	0.02
Long Covid	8.08	<0.001	0.06	7.22	<0.001	0.06	3.06	0.05	0.02	0.57	0.57	0.004
Model *R^2^*	0.26			0.22			0.20			0.18		

Table 3 shows results of the post hoc tests examining differences between long covid groups on mental and physical health. The self‐reported long covid group had significantly greater anxiety and depression than the no long covid group. The diagnosed long covid group had significantly greater physical function problems than the no long covid group. Both long covid groups had significantly greater fatigue, social roles/activity limitations, and pain interference than the no long covid group.

**TABLE 3:** Post hoc follow‐up comparisons of long covid group on mental and physical health among University of Southern California Patients in Los Angeles, CA, 2023.

	Diagnosed long covid	Self‐reported long covid	No long covid
Anxiety	1.89^ab^	2.16^a^	1.69^b^
Depression	1.70^ab^	2.06^a^	1.56^b^
Physical function	2.15^a^	1.92^ab^	1.64^b^
Fatigue	3.11^a^	3.13^a^	2.31^b^
Social roles/Activity limitations	2.60^a^	2.40^a^	1.94^b^
Pain interference	2.59^a^	2.36^a^	1.92^b^
Poor sleep	2.86^a^	2.96^a^	2.63^a^
Eating disorder symptoms	1.68^a^	1.78^a^	1.70^a^

*Note*. Means with different superscripts are significantly different (*p* < 0.05).

## DISCUSSION

The COVID‐19 pandemic had a widespread effect on global physical and mental health [25, 26], and long covid represents a long‐term challenge that emerged from the virus. The present study aimed to investigate associations of long covid (diagnosed and self‐reported) with mental and physical health, controlling for key covariates. Findings showed important differences between long covid groups on mental and physical health.

Consistent with a recent meta‐analysis [10], participants with diagnosed or self‐reported long covid had elevated fatigue compared to those without long covid, showing a medium‐to‐large effect size. In addition, both long covid groups had significantly greater social roles/activity limitations and pain interference than the no long covid group, which aligns with other research on pain and activity limitations in long covid [27, 28]. The diagnosed long covid group also had significantly greater physical function problems than the no long covid group, but the self‐reported long covid group did not differ from the other groups. It is possible that elevated physical function problems (e.g., not being able to do daily activities) may contribute to a higher likelihood of receiving a long covid diagnosis.

Participants with self‐reported long covid had significantly greater anxiety and depression than the no long covid group; however, there were no other group differences. Studies have shown that long covid is associated with anxiety, depression, and stress [29], potentially due to the uncertainty surrounding the condition and symptoms. Given that those diagnosed with long covid did not show elevated anxiety and depression, it is possible that receiving a diagnosis helps alleviate the mental health burden of long covid, including reducing intolerance of uncertainty [8]. Receiving a diagnosis and understanding the underlying cause of their physical symptoms may help patients stop the cycle by which the unknown nature of their physical symptoms leads to anxiety [30]. Moreover, receiving a diagnosis may lead to the start of treatment and the potential alleviation of symptoms [31]. However, it is possible that the presence of anxiety and depressive symptoms is associated with less likelihood of a formal diagnosis of long covid being given. Importantly, Hispanic participants had elevated rates of having self‐reported long covid, which may be due to several factors such as reduced healthcare coverage or structural racism in healthcare often experienced by minorities [32, 33]. Inequities have been discussed and are still emerging in addressing long covid among people of color [34]. Failure to address long covid symptoms in Hispanic individuals could lead to increased mental healthcare burden for Hispanics over time.

Alternatively, there is also evidence of bidirectional associations between mental health and long covid symptoms, with depression and anxiety being risk factors for long covid and long covid leading to poor mental health [35]. Given elevated mental health problems only among the self‐report long covid group and not the diagnosed group, it is possible those with prior mental health challenges are more aware of their body/health as it relates to chronic conditions. Therefore, if they have lasting symptoms, they might be more likely to report having long covid even though they might not be diagnosed with long covid.

COVID‐19 distress was associated with poor outcomes, with higher effect sizes for mental health outcomes. Individuals with long covid reported more COVID‐19 distress, and thus, there could be synergistic effects of these factors in predicting mental and physical health outcomes. However, models showed that COVID‐19 distress had an independent effect on outcomes controlling for long covid, especially with regard to mental health outcomes; this underscores the importance of assessing and treating COVID‐19 distress in addition to long covid.

Although research is still emerging, there are several biologic and psychosocial interventions that have been proposed for treating long covid [36, 37]. However, individuals with long covid symptoms have reported having low access to services and treatments, and this lack of access has led to stress and anxiety [38]. In addition, when accessing treatment services, many individuals with long covid symptoms report experiencing discrimination and stigma from healthcare professionals, including not believing symptoms [39]. Although long covid is complex and can manifest in many different ways, it is critical to provide evidence‐based approaches to the assessment and treatment of long covid [40]. In addition, a trauma‐informed approach has been recommended given the traumatic nature of a life‐altering chronic condition [40].

Although this study adds to our knowledge of long covid and mental and physical health, there are several limitations that must be noted. All data were self‐reported by participants, which is subject to recall biases. The study recruited adults from a clinical database in Southern California, which may not be representative or generalizable to other people in the United States or globally. Preexisting history of psychiatric disorders prior to COVID‐19 infection may be an important factor in understanding the effect of long covid on mental and physical health [41], but prior psychiatric history was not collected. Further, post‐traumatic stress disorder symptoms have been associated with long covid but were not measured in the current study [42]. Moreover, the measure of COVID‐19 distress was validated among data collected from adults in 2020–2021 [23], and it is unclear if there are differences in acute versus chronic pandemic‐related distress that are not captured by this measure. Finally, this study was cross‐sectional, and thus, causal interpretations cannot be elucidated from this data.

## CONCLUSION

In summary, this study provides insights into the association of long covid with aspects of mental and physical health. Findings highlighted fatigue as the most significant symptom associated with long covid, with other physical health challenges reported as well. In addition, negative mental health was only experienced by individuals with self‐reported long covid, suggesting the importance of long covid diagnosis and treatment. Addressing mental and physical health symptoms associated with long covid is critical to reducing the long‐term health burden of this condition. Research will be needed to determine interventions that improve physical and mental health outcomes, especially symptoms of fatigue.

## AUTHOR CONTRIBUTIONS

*Conceptualization; data curation; formal analysis; funding acquisition; investigation; methodology; writing—original draft*: Tyler B. Mason. *Data curation; methodology; writing—reviewing and editing*: Tara K Knight. *Data curation; methodology; software; writing—reviewing and editing*: Ryan Lee and Shirin E. Herzig. *Conceptualization; funding acquisition; investigation; methodology; writing—reviewing and editing*: Daniella Meeker and Jason N. Doctor.

## CONFLICT OF INTEREST STATEMENT

The authors have no conflicts of interest.

## FUNDING INFORMATION

This project was supported by a grant from the USC Institute for Health Promotion & Disease Prevention Research (IPR).

## ETHICS STATEMENT

All study procedures were reviewed and approved by the USC Institutional Review Board (HS‐20‐00793).

## PATIENT CONSENT

Informed consent was obtained through e‐consent in REDCap.

## Data Availability

Data is available through contacting the corresponding author.
